# The impact of sarcopenia on prognosis and fruquintinib efficacy in advanced colorectal cancer: a retrospective and mendelian randomization study

**DOI:** 10.3389/fimmu.2025.1582308

**Published:** 2025-07-16

**Authors:** Yao Wang, Du Meilu, Shi Qi, Tang Yufei, Gao Song, Han Susu, Wen Haotian, Zhu Tingting, Wang Chao, Hou Fenggang, Yin Xiaoling

**Affiliations:** ^1^ Department of Oncology, Unit 3, Shanghai Municipal Hospital of Traditional Chinese Medicine, Shanghai, China; ^2^ Department of Oncology, Ruijin Hospital, Shanghai Jiao Tong University School of Medicine, Shanghai, China

**Keywords:** sarcopenia, immune, nutrition, advanced colorectal cancer, fruquintinib, mendelian randomization study

## Abstract

**Background:**

Reportedly, sarcopenia is associated with prognosis in advanced colorectal cancer (CRC) patients and can lead to reduced efficacy of targeted therapy. However, studies on the relationship between sarcopenia and the prognosis (or efficacy) of advanced CRC patients receiving fruquintinib targeted therapy remains scarce. Therefore, we conducted a comprehensive assessment of the relationship between nutritional status, inflammation, immune function, and cancer-related sarcopenia. We also investigated whether sarcopenia affects the therapeutic efficacy of fruquintinib targeted therapy.

**Patients and methods:**

In this retrospective study, sarcopenia and several markers of nutritional status and immune function were assessed in advanced CRC patients with fruquintinib therapy at the hospital. We used drug target mendelian randomization (MR) analysis to investigate the impact of fruquintinib on sarcopenia.

**Results:**

Advanced CRC patients with sarcopenia had a poorer prognosis compared to those without sarcopenia. Furthermore, sarcopenia showed a strong correlation with various markers of nutritional status, immune function indicators, inflammation markers, quality of life scores, and the prognostic nutrition index. MR studies suggest that the spleen tyrosine kinase (SYK) gene is a key factor in the occurrence of sarcopenia associated with the use of fruquintinib.

**Conclusion:**

Sarcopenia could be a prognostic factor in patients with advanced CRC receiving fruquintinib targeted therapy.

## Introduction

Colorectal cancer (CRC) poses significant health challenges worldwide, with alarming statistics reflecting its severity. The five-year survival rate for patients with metastatic CRC stands at approximately 14% (1), underscoring the dire prognosis for this condition. Epidemiological data indicates that CRC ranks as the third most common and lethal type of cancer globally. In 2020, it was estimated that there were over 1.9 million new cases and nearly 1 million deaths attributed to this malignancy (2), highlighting the urgent need for enhanced prevention, early detection, and effective treatment strategies (3).

The etiology of CRC is multifactorial, intricately linked to genetic, environmental, and lifestyle factors, making early diagnosis essential for improving patient outcomes (4–6). The low 5-year overall survival rate in CRC is primarily due to the metastasis of CRC (7, 8). The contemporary clinical management of CRC involves a multidisciplinary approach that integrates surgical intervention, chemotherapy, targeted therapy, and immunotherapy (9, 10). Patients with tumors frequently experience a state of metabolic dysregulation, which is typified by increased catabolic processes and diminished anabolic activity (11, 12). Sarcopenia, a syndrome characterized by the progressive loss of skeletal muscle mass and function, is increasingly recognized as a significant factor affecting the prognosis of cancer patients, particularly those with CRC (13, 14). Sarcopenia can lead to metabolic disorders, along with inflammatory reactions and immune dysfunction (15–17). Recently, fruquintinib, an innovative targeted therapy for advanced CRC, has shown promise as a third-line treatment option, offering potential survival benefits for these patients (18).

The prevalence of muscle wasting among advanced CRC patients, due to impaired digestive and absorptive capacity, raises concerns regarding its impact on the efficacy of targeted therapies. In a study of colorectal cancer patients, sarcopenia was found in 18.8% of the cohort and linked to poorer progression-free and overall survival (19). A prior study demonstrated the prognostic significance of baseline skeletal muscle index (SMI) in patients with metastatic CRC undergoing treatment with fruquintinib (20). However, the impact of sarcopenia on the therapeutic efficacy of fruquintinib, a widely utilized novel targeted therapy, remains uncertain. This study aims to investigate the impact of sarcopenia on patients with advanced CRC patients undergoing targeted therapy through a retrospective analysis. Addtionally, it seeks to identify key genes between fruquintinib and sarcopenia using MR analysis. Specifically, we hypothesize that sarcopenia serves as a prognostic factor in advanced CRC patients undergoing fruquintinib treatment, and identifying the key genes associated with fruquintinib-induced sarcopenia is crucial for elucidating its underlying mechanisms and optimizing therapeutic outcomes.

## Methods

### Patients and study design

In this retrospective study, patients treated for advanced CRC using fruquintinib, were selected from January 2021 to January 2024 at the Shanghai Municipal Hospital of Traditional Chinese Medicine.

### Measurement of muscle mass

**Figure 1:**
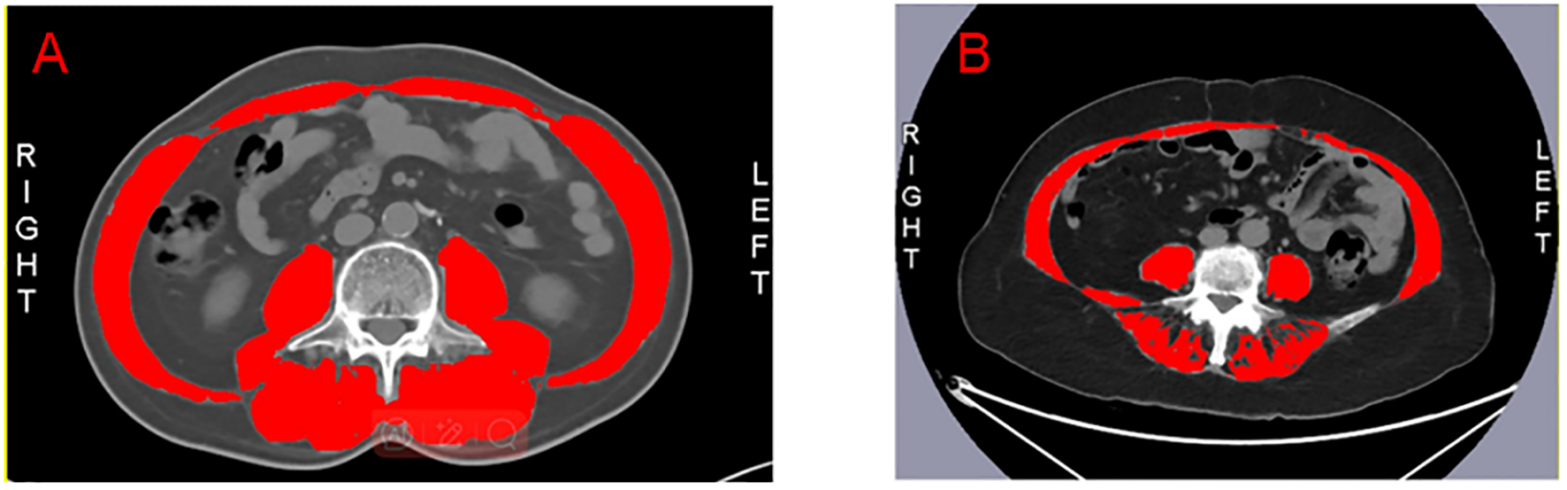
The red section represents the skeletal muscle at the L3 level. **(A)** The L3 level in patients with non-sarcopenia. **(B)** The L3 level in patients with sarcopenia.

As previously outlined, sarcopenia assessment follows a method summarized below (21). Muscle mass was quantified through cross-sectional computed tomography (CT) imaging at the third lumbar (L3) level, measuring the skeletal muscle area using hounsfield units (HU) ranging from –29 to 150. We use the Slice Omatic 5.0 image analysis software to measure the L3 level. The SMI was calculated by normalizing the muscle area to height (cm²/m²), SMI is indicative of sarcopenia if less than 40.31 cm²/m² in males and 30.88 cm²/m² in females (22). Prior to fruquintinib treatment, CRC patients underwent standardized CT evaluations, categorizing them into sarcopenia groups (SG) and non-sarcopenia groups (NSG) based on SMI values, thus enabling precise assessment of sarcopenia’s impact on treatment outcomes. **Figure 1** demonstrates the comparison between CRC patients with sarcopenia (**Figure 1A**) and those without sarcopenia (**Figure 1B**), as visualized by CT scans at the L3 level, highlighting the skeletal muscle area.

### Nutritional status assessment

We employed various traditional nutritional assessments, such as body mass index (BMI), SMI, albumin (ALB), prealbumin (PAB), retinol-binding protein (RBP) and the prognostic nutrition index (PNI).

### Inflammatory status assessment

We assessed inflammatory markers, including interleukin-1β (IL-1β), interleukin-2 (IL-2), interleukin-6 (IL-6), interleukin-12 (IL-12), tumor necrosis factor-α (TNF-α), and interferon-γ (IFN-γ). These markers were measured using was conducted using a fully automated flow cytometer (NovoCyte D2060R).

### Immune status assessment

We assessed immune markers, including the absolute counts of CD3, CD4, CD8, and natural killer (NK) cells. These immune markers were analyzed using a fully automated flow cytometer (BD FACSCanto II).

Statistical analysis was performed using SPSS (version 29). For continuous variables that followed a normal distribution, data are presented as mean ± standard deviation (SD), and group differences were analyzed using the t-test. For continuous variables that did not follow a normal distribution, data are presented as median (interquartile range), and group differences were analyzed using the Wilcoxon test. Categorical variables are described using frequency (percentage), and group differences were compared using the chi-square test or Fisher’s exact test. To evaluate the relationships between variables, we used spearman correlation analysis, which measures the strength and direction of monotonic associations and is suitable for non-parametric data and ordinal variables.

### Ethical consideration

The protocol for this investigation was approved by the Shanghai Municipal Hospital of Traditional Chinese Medicine (2021SHL-KY-04-03).

### Study design of MR

To explore the causal relationship between fruquintinib and sarcopenia, we conducted a MR study. We validated the reliability of genetic variants as instrumental variables (IVs) by fulfilling three key MR assumptions. Firstly, we selected genome-wide significant variants (*P* < 5×10^-8^) strongly linked to the exposure, ensuring F-statistics > 10 to avoid weak instrument bias (20). Secondly, we screened IVs for associations with potential confounders (age, sex, BMI, smoking status, socioeconomic status) using PhenoScanner and multivariable MR adjustment. Thirdly, we confirmed that the IVs affect the outcome only through the exposure, with horizontal pleiotropy tests (MR-Egger intercept and MR-PRESSO) showing all *P* > 0.05 (23). In this study, gene expression quantitative trait loci (eQTLs) were employed as exposure variables, whereas indicators associated with left-hand grip strength, right-hand grip strength, appendicular muscle mass, and walking speedwere considered as outcome variables. **Supplementary Figure S1** displays the study flowchart.

### Sources

Three genes were identified from DrugBank and seven from the DGIdb database for fruquintinib. The SMILES structures of the compounds were retrieved from the PubChem database (https://pubchem.ncbi.nlm.nih.gov/). Target gene prediction using SwissTargetPrediction (http://www.swisstargetprediction.ch/) yielded 117 potential target genes. After merging and deduplicating results from the three databases, a final list of 111 target genes for fruquintinib was established.

The diagnosis of sarcopenia was referred to the criteria established by the European Working Group on Sarcopenia in Older People (EWGSOP) (24). This study includes 461,089 European samples with 9,851,867 single nucleotide polymorphisms (SNPs) associated with grip strength, including both left and right hand grip strength. For appendicular lean mass, 450,243 European samples and 18,071,518 SNPs were analyzed. Usual walking pace is associated with 459,915 European samples and 9,851,867 SNPs. A detailed description of the data used in this study is provided in **Supplementary Table S1**.

### Statistical analysis of MR

To address horizontal pleiotropy in MR analyses, MR-PRESSO detected no outliers with Distortion test *P*-values < 0.05. Five methods—IVW, MR-Egger, Weighted Median, Simple Mode, and Weighted Mode (25)—were applied, prioritizing IVW results. Heterogeneity tests guided the use of random effects (*P* < 0.05) or fixed effects (*P* > 0.05) in IVW. The robustness of the results was ensured through sensitivity analyses, which included heterogeneity testing using mr_heterogeneity(), the application of MR-Egger regression to assess pleiotropy, and the implementation of a leave-one-out analysis (26). Causal effects were reported as odds ratios (OR) with 95% confidence intervals (CI). Analyses were performed using R packages “TwoSampleMR” (v 0.6.0) and “MRPRESSO” (v 1.0).

## Results

### Patients’ characteristics

Of the 60 eligible patients, 31 were categorized into the sarcopenia group and 29 into the non-sarcopenia group. **Table 1** details patients’ background.

**Table 1:** Patients’ basic characteristics.

Variables	Total (n = 60)	Sarcopenia (n = 31)	Non-sarcopenia (n = 29)	*P*-Value
Sex, n (%)				0.76
Female	35 (58.33)	17 (54.84)	18 (62.07)	
Male	25 (41.67)	14 (45.16)	11 (37.93)	
Age, Mean ± SD (years)	64.47 ± 10.87	66.84 ± 8.9	61.93 ± 12.29	0.172
Hight, Median (Q1,Q3) (m)	1.63 (1.59, 1.71)	1.63 (1.6, 1.71)	1.63 (1.58, 1.7)	0.888
Weight, Median (Q1,Q3) (kg)	60 (55, 66)	57 (53.5, 65)	60 (55, 68)	0.029*
BMI, Mean ± SD (kg/m²)	22.38 ± 2.91	21.08 ± 2.56	23.77 ± 2.64	< 0.001***
SMI, Mean ± SD (cm²/m²)	39.68 ± 8.77	33.73 ± 4.7	46.03 ± 7.59	< 0.001***
ECOG score, n (%)				0.07
0	12 (20)	3 (9.68)	9 (31.03)	
1	35 (58.33)	18 (58.06)	17 (58.62)	
2	6 (10)	4 (12.9)	2 (6.9)	
3	7 (11.67)	6 (19.35)	1 (3.45)	
Primary site, n (%)				0.408
Transverse colon	1 (1.67)	0 (0)	1 (3.45)	
Cecum and ileocecal region	3 (5)	2 (6.45)	1 (3.45)	
Descending colon	2 (3.33)	0 (0)	2 (6.9)	
Ascending colon	5 (8.33)	2 (6.45)	3 (10.34)	
Sigmoid colon	29 (48.33)	18 (58.06)	11 (37.93)	
Rectum	20 (33.33)	9 (29.03)	11 (37.93)	
Pulmonary metastasis, n (%)				0.846
No	48 (80)	24 (77.42)	24 (82.76)	
Yes	12 (20)	7 (22.58)	5 (17.24)	
Hepatic metastasis, n (%)				1
Yes	60 (100)	31 (100)	29 (100)	
Bone metastasis, n (%)				0.492
No	58 (96.67)	29 (93.55)	29 (100)	
Yes	2 (3.33)	2 (6.45)	0 (0)	
Brain metastasis, n (%)				1
No	59 (98.33)	30 (96.77)	29 (100)	
Yes	1 (1.67)	1 (3.23)	0 (0)	
Chemotherapy, n (%)				1
No	3 (5)	2 (6.45)	1 (3.45)	
Yes	57 (95)	29 (93.55)	28 (96.55)	
Surgery, n (%)				1
No	3 (5)	2 (6.45)	1 (3.45)	
Yes	57 (95)	29 (93.55)	28 (96.55)	
Targeted therapy, n (%)				0.815
No	27 (45)	13 (41.94)	14 (48.28)	
Yes	33 (55)	18 (58.06)	15 (51.72)	
Immunotherapy, n (%)				0.053
No	55 (91.67)	26 (83.87)	29 (100)	
Yes	5 (8.33)	5 (16.13)	0 (0)	

BMI, Body Mass Index; SMI, Skeletal Muscle Index; ECOG, Eastern Cooperative Oncology Group performance status. *P < 0.05, ***P < 0.001.

### Comparison of nutritional, inflammatory, and immune indexes

Previous study found that sarcopenia correlates with the nutrition indicators (27). We assessed the relationship between nutritional markers such as albumin, prealbumin, retinol-binding protein, and sarcopenia in our study. **Table 2** presents that compared to the NSG, patients in the SG had significantly lower levels of albumin, prealbumin, and retinol-binding protein. As a proinflammatory cytokine, IL-6, along with IL-6 signaling, plays a crucial role in the inflammatory microenvironment linked to CRC (28, 29). Furthermore, the progression of sarcopenia is associated with elevated serum levels of IL-6 (30). We found that IL-6 level was significantly higher in the SG than in the NSG (*P* < 0.05). Sarcopenia may impair myokine signaling, shift membrane-bound factors to a pro-inflammatory state, and reduce immune cell regeneration, leading to immune dysfunction (31). We compared absolute counts of CD3, CD4, CD8, and NK cells between the two groups. Our results revealed that, compared to the NSG, patients in the SG had a significant decrease in the absolute count of NK cells (*P* < 0.01). Thus, it is evident that tumor-associated sarcopenia may lead to alterations in the patient’s nutritional, inflammatory, and immune status.

**Table 2:** Analysis of the inter-group differences for each diagnostic parameter.

Variables	Sarcopenia (n = 31)	Non-sarcopenia (n = 29)	*P*-Value
AlB (g/L)	34.78 ± 5.34	39.81 ± 5.14	<.001**
PAB (mg/L)	190.1 ± 67.00	258.34 ± 67.00	<.001**
RBP (mg/L)	28.19 ± 11.41	37.02 ± 9.83	0.002**
IL-1β (pg/mL)	3.23 (1.35~5.97)	3.6 (1.745~5.22)	0.559
IL-2 (pg/mL)	5.13 (3.27~8.08)	4.24 (2.93~7.355)	0.416
IL-6 (pg/mL)	8.13 (2.83~12.9)	4.52 (2.605~6.38)	0.031*
IL-12 (pg/mL)	3.67 (1.81~4.83)	2.36 (1.36~4.505)	0.277
TNF-α (pg/mL)	2.69 (1.91~5.63)	2.9 (1.875~4.875)	0.97
IFN-γ (pg/mL)	1.8 (0.9~2.9)	1.9 (0.85~4.4)	0.455
CD3 (/μL)	804 (538~1165)	891 (684~1152)	0.171
CD4 (/μL)	477 (245~668)	609 (340~808.5)	0.115
CD8 (/μL)	288 (173~434)	280 (219~441)	0.363
NK (/μL)	123 (78~177)	229 (159.5~323.5)	<.001**
PNI (g/dL)	40.54 ± 6.99	47.12 ± 5.85	0.219

ALB, Albumin; PAB, Prealbumin; RBP, Retinol-binding protein; IL-1β, Interleukin-1β; IL-2, Interleukin-2; IL-6, Interleukin-6; IL-12, Interleukin-12; TNF-α, Tumor Necrosis Factor-α; IFN-γ, Interferon-γ; CD3, Cluster of Differentiation 3; CD4, Cluster of Differentiation 4; CD8, Cluster of Differentiation 8; NK, Natural Killer Cell; PNI, Prognostic nutritional index. *P < 0.05, **P < 0.01.

### Correlation analysis

**Figure 2:**
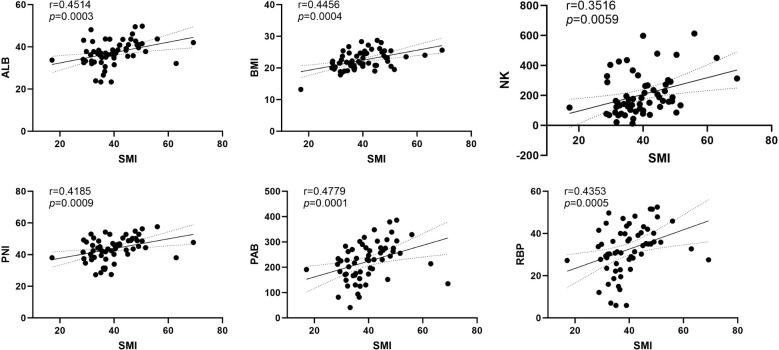
The correlation analysis between SMI and ALB, PAB, RBP, NK, BMI and PNI. ALB, Albumin (g/L); PAB, Prealbumin (mg/L); RBP, Retinol-binding protein (mg/L); NK, Natural Killer Cell (/μL); BMI, Body Mass Index (kg/m²); PNI, Prognostic nutritional index (g/dL).

Subsequently, Our correlation analysis demonstrated a significant association between sarcopenia and several biomarkers, including albumin, prealbumin, retinol-binding protein, NK cells, BMI, and PNI. Based on these results, sarcopenia showed the strongest correlation with nutritional markers overall (**Figure 2**).

### Efficacy for all patients

**Figure 3:**
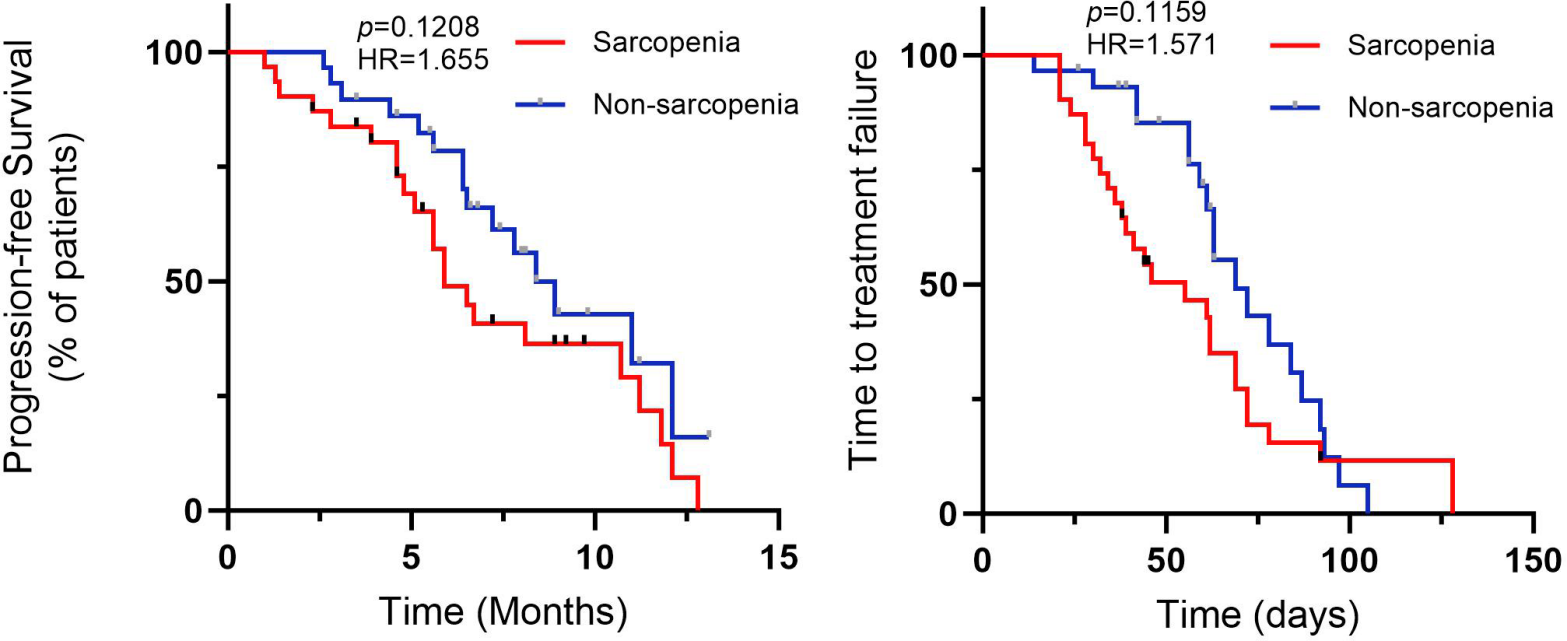
Profession-free survival (*PFS*), and time to treatment failure (*TTF*) for CRC patients with fruquintinib therapy.

In SG the median progression-free survival (*PFS*) was 5.9 months (95% CI=0.3644-1.454, HR=1.655). In NSG the median PFS was 8.4 months (95% CI=0.71386-2.744, HR=0.6042). The median time to treatment failure (*TTF*) in SG was 59 days (95% Cl=0.4412-1.440, HR=1.571) while in NSG this value was 69 days (95% Cl=0.6943-2.267, HR=0.6364). **Figure 3** demonstrates the Kaplan-Meier curves of PFS and TTF. In terms of PFS and TTF, no significant differences were observed between SG and NSG.

### Safety analysis

**Figure 4:**
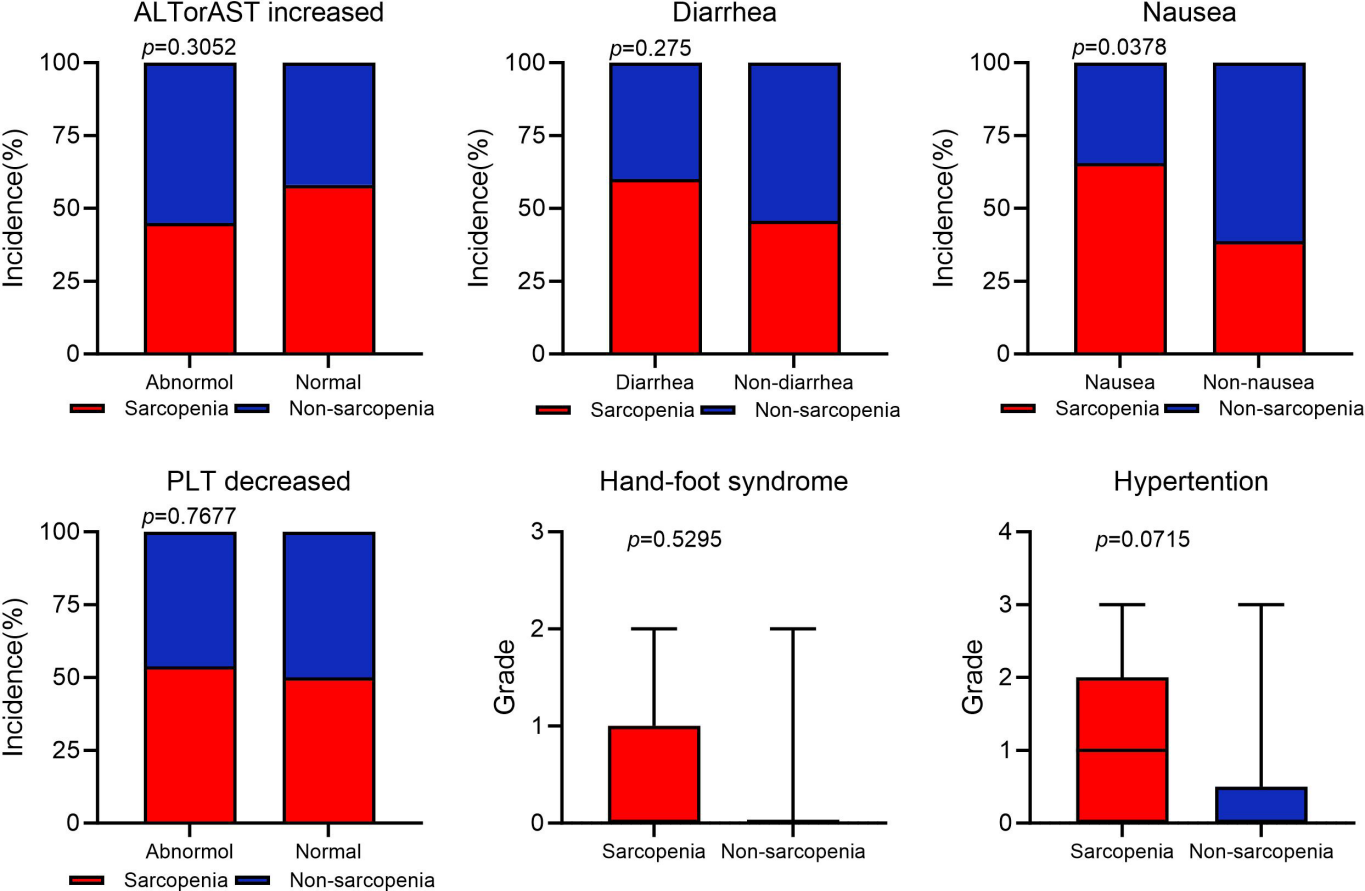
Adverse event occurrence statistics. AST, aspirate aminotransferase (U/L); ALT, alanine aminotransferase (U/L); PLT, platelet (×10^9^/L).

According to reports, the main side effects of fruquintinib include hypertension, gastrointestinal reactions such as nausea, vomiting, and diarrhea, abnormal bleeding such as epistaxis and hematuria, as well as hand-foot syndrome and abnormalities in liver and kidney function (32–34). We analyzed the occurrence of adverse effects during fruquintinib targeted therapy in patients with and without sarcopenia (**Figure 4**). Our results indicated that patients in the SG presented the higher incidence of nausea than in the NSG (*P*<0.05). Patients in the SG exhibited higher levels of hypertension, hand-foot syndrome, and severity of symptoms compared to the NSG. Additionally, the SG had a higher incidence of diarrhea, and thrombocytopenia. However, there were no significant inter-group differences in these indicators.

### MR results between the target genes of fruquintinib and left-hand grip strength

The relationship between the target genes of fruquintinib and left-hand grip strength are illustrated in **Table 3**. A *p*-value < 0.05 in MR analysis signifies a significant causal link. If the OR is > 1, it suggests that the gene is a risk factor for the outcome, meaning that an increase in the expression level of this gene is associated with a higher risk of the outcome. Conversely, if the OR is < 1, the gene is considered a protective factor, indicating that higher expression levels of this gene are associated with a decreased risk of the outcome. A total of 28 genes exhibited *P-*values < 0.05 between the target genes of fruquintinib and left-hand grip strength. The following drug target genes are correlated with an elevated risk (OR > 1) of adverse outcomes: FLT4, CA2, ZAP70, HDAC1, ADORA2A, SLC27A1, CDK7, CTSL, CTSK, CTSS, ADORA1, SYK, IMPDH2, IRAK4, MTOR.

**Table 3:** MR results of exposure factors and outcomes (left hand grip strength).

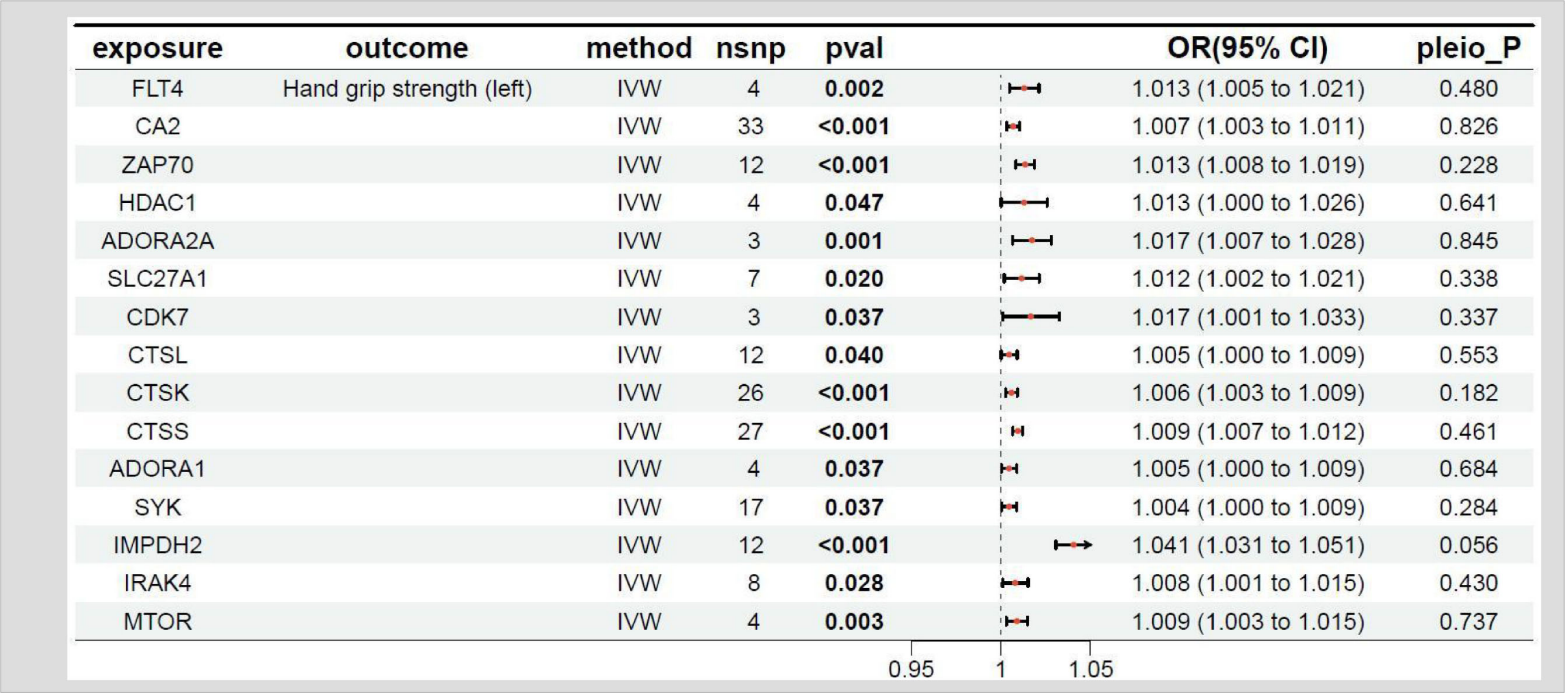

pavl: Significance; OR, Odds ratio, with the 95% confidence interval of the odds ratio in parentheses; pleio_P: Test for horizontal pleiotropy.

### MR results between the target genes of fruquintinib and right-hand grip strength

The relationship between the target genes of fruquintinib and right-hand grip strength are illustrated in **Table 4**. A total of 25 genes exhibited *P-*values < 0.05. The following drug target genes are associated with an increased risk of adverse outcomes: FLT4, CA2, ZAP70, HDAC1, PTK2B, PTGFR, ADORA2A, CDK7, CTSL, NAAA, CTSS, SYK, PTGER4, MTOR.

**Table 4:** MR results of exposure factors and outcomes (right hand grip strength).

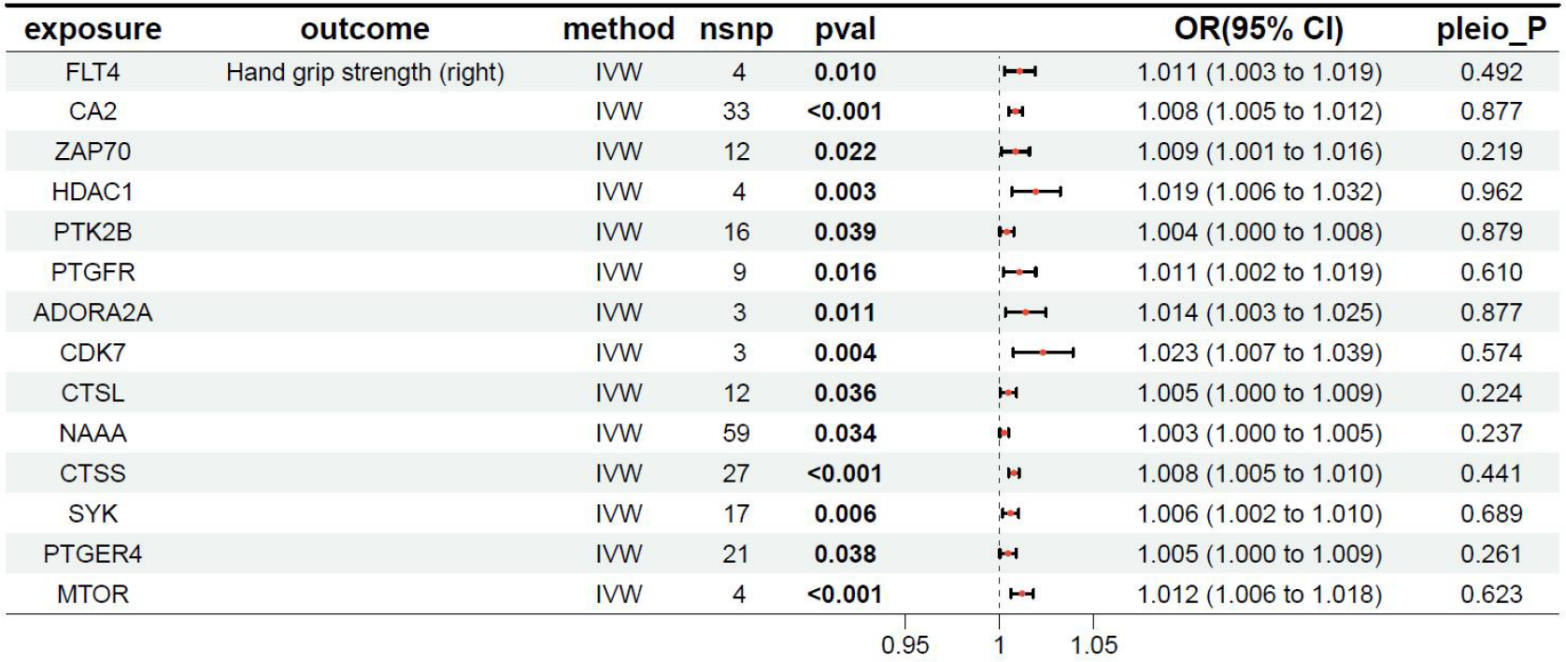

pavl: Significance; OR, Odds ratio, with the 95% confidence interval of the odds ratio in parentheses; pleio_P: Test for horizontal pleiotropy.

### MR results between the target genes of fruquintinib and muscle mass

The relationship between the target genes of fruquintinib and limb muscle mass are illustrated in **Table 5**. A total of 21 genes exhibited *P-*values < 0.05. The following drug target genes are associated with an increased risk of adverse outcomes: BAD, FLT4, CDC7, PDE8B, ZAP70, PTK2B, CDK7, CTSL, CTSK, CTSS, FNTA, MTOR.

**Table 5:** MR results of exposure factors and outcomes (limb muscle mass).

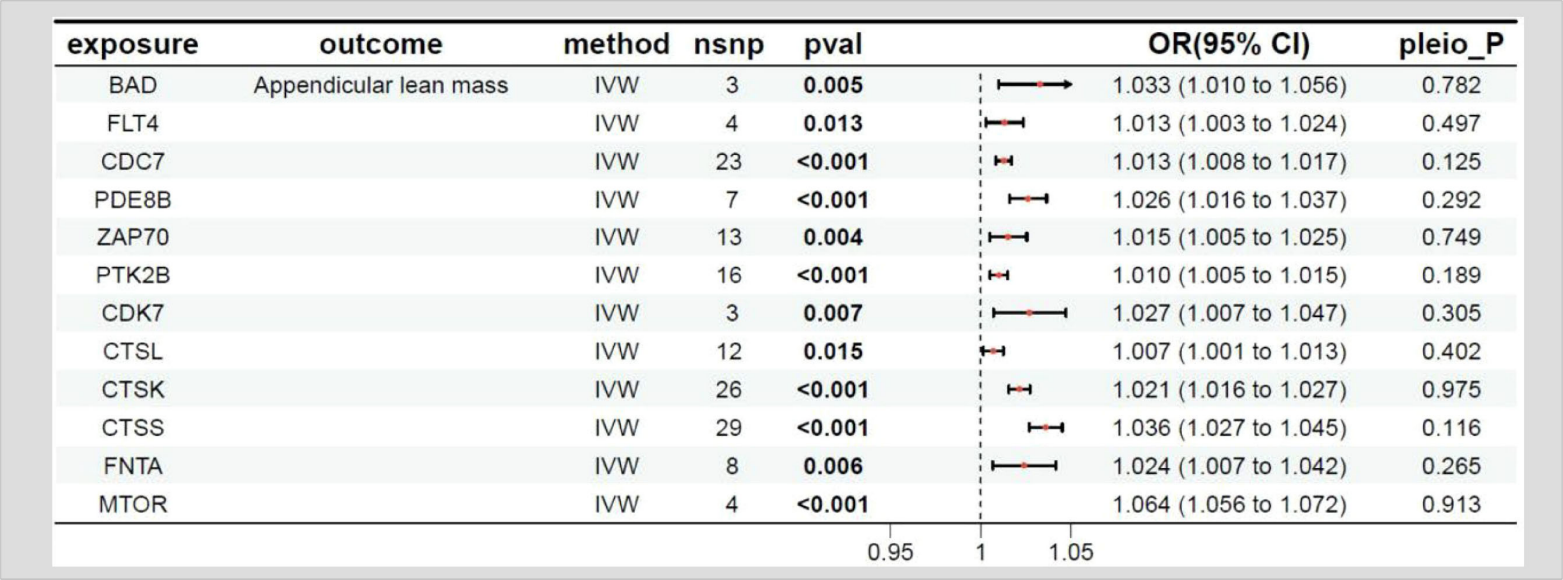

pavl: Significance; OR, Odds ratio, with the 95% confidence interval of the odds ratio in parentheses; pleio_P: Test for horizontal pleiotropy.

### MR results between the target genes of fruquintinib and walking speed

The relationship between the target genes of fruquintinib and walking speed are illustrated in **Table 6**. A total of 12 genes exhibited *P-*values < 0.05. The following drug target genes are associated with an increased risk of adverse outcomes: BAD, FGFR2, HSP90AA1, PDE8B, PTK2B, SYK, STAT3.

**Table 6:** MR results of exposure factors and outcomes (walking speed).

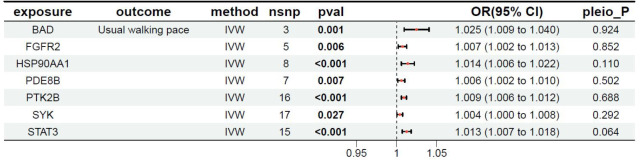

pavl: Significance; OR, Odds ratio, with the 95% confidence interval of the odds ratio in parentheses; pleio_P: Test for horizontal pleiotropy.

The intersection of MR results for drug target genes and the four outcome variables mentioned above was visualized, as shown in **Supplementary Figure S2**. Two genes, the BCL2 associated agonist of cell death (BAD) gene and the spleen tyrosine kinase (SYK) gene, were found to have a significant causal relationship with all four outcome variables (**Table 7**). The SYK gene is considered to be the most likely candidate gene associated with an increased risk of sarcopenia.

**Table 7:** The significant results of the four outcome variables.

Exposure	Outcome	Id.exposure	Id.outcome	Method	Nsnp	Pval	Or
BAD	Hand grip strength (left)	eqtl-a-ENSG00000002330	ukb-b-7478	IVW	3	8.83E-10	0.946
	Hand grip strength (right)		ukb-b-10215	IVW	3	6.74E-04	0.970
	Appendicular lean mass		ebi-a-GCST90000025	IVW	3	0.005	1.033
	Usual walking pace		ukb-b-4711	IVW	3	0.001	1.025
SYK	Hand grip strength (left)	eqtl-a-ENSG00000165025	ukb-b-7478	IVW	17	0.037	1.004
	Hand grip strength (right)		ukb-b-10215	IVW	17	0.006	1.006
	Appendicular lean mass		ebi-a-GCST90000025	IVW	17	0.003	0.992
	Usual walking pace		ukb-b-4711	IVW	17	0.027	1.004

**Figure 5:**
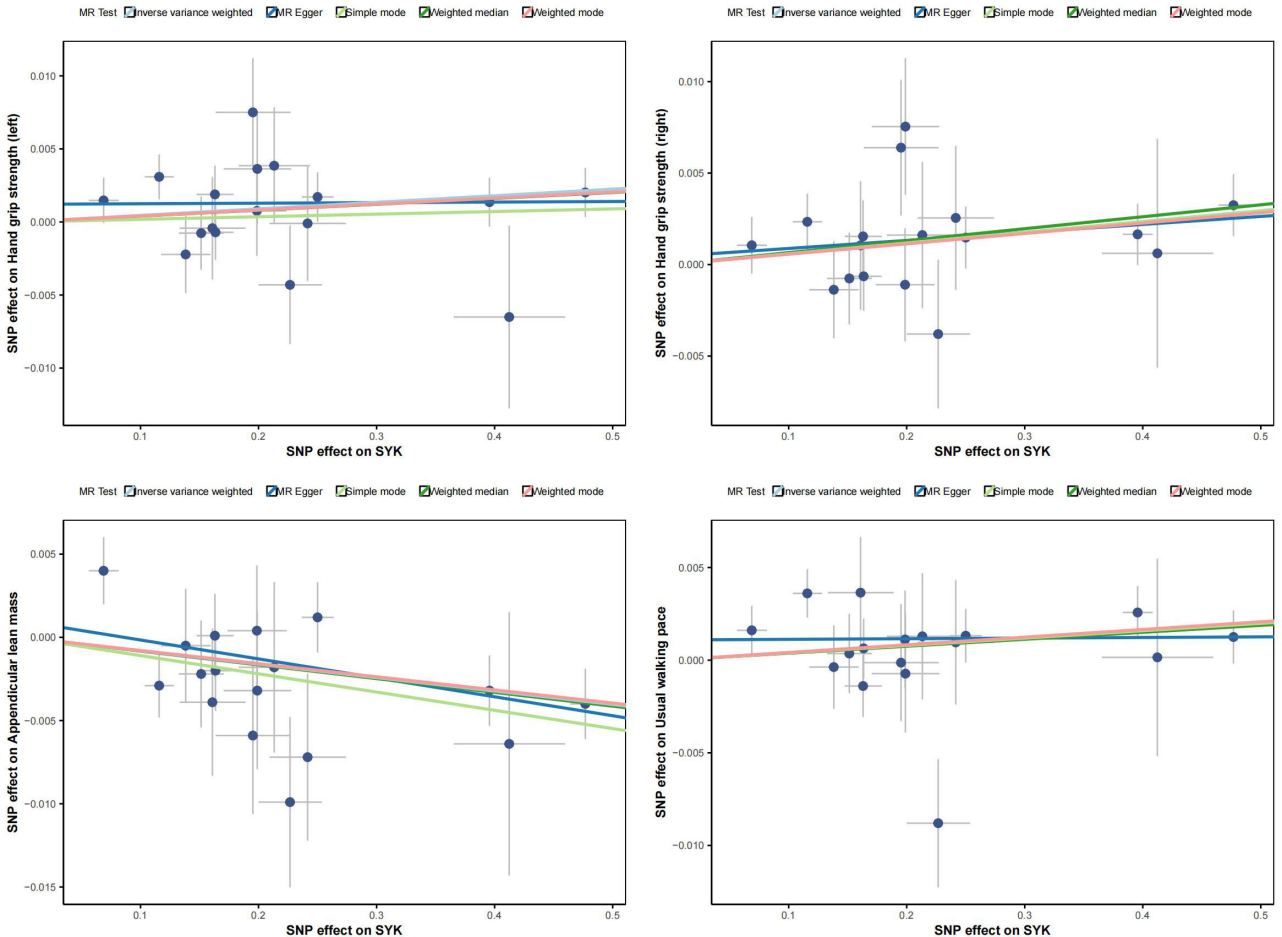
Scatter plot of exposure factors and outcomes.

**Figure 6:**
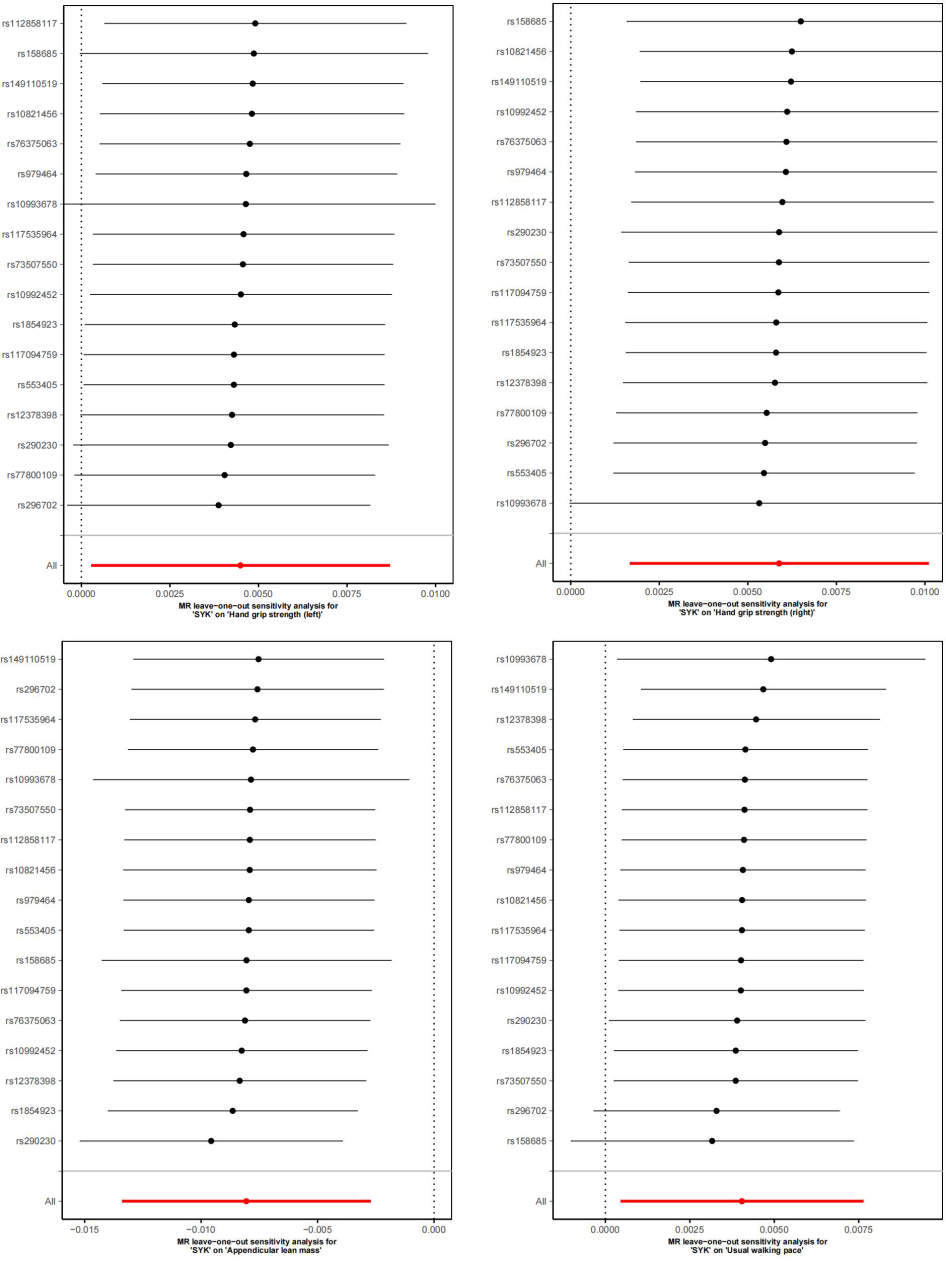
Forest plot for the sequential exclusion of exposure factors and outcomes.

**Figure 5** displays scatter plots of the associations between exposure factors and outcome variables obtained from five MR algorithms. The leave-one-out sensitivity analysis, shown in **Figure 6**, demonstrates that the removal of each SNP individually does not significantly affect the outcome variable, indicating that the results of this MR analysis are reliable and stable.

## Discussion

This study provides significant insights into the relationship between sarcopenia and the clinical outcomes of fruquintinib targeted therapy in advanced CRC patients. Recently, poor baseline nutrition has been viewed as a predictor of cancer outcomes, particularly in gastrointestinal tumors (35, 36). A decline in SMI, BMI, and nutritional markers, including albumin, prealbumin, and the PNI, may serve as indicators for assessing the risk of sarcopenia (37–39). Moreover, the presence of sarcopenia can diminish the effectiveness of tumor-targeted therapies (40, 41). We observed that sarcopenic patients exhibited a significant decline in nutritional markers, including albumin, prealbumin, and retinol-binding protein, in comparison to non-sarcopenic patients (**Table 2**). Studies have shown that IL-6 levels correlate with muscle wasting and atrophy, which are key features of sarcopenia (42, 43). IL-6 is a cytokine that plays a significant role in inflammation and the immune response (44). In particular, IL-6 can suppress the function of NK cells, leading to a reduced ability to target and eliminate cancer cells (45, 46). Patients with sarcopenia exhibited increased concentrations of IL-6 and a significant decrease in NK cell counts, indicating a potential impairment in immune function (**Table 2**). Sarcopenic patients had a shorter duration of fruquintinib treatment and faced more adverse events, particularly gastrointestinal symptoms like nausea, and diarrhea. These symptoms can decrease appetite and food intake, worsening nutritional status and nutrient absorption, which in turn aggravates sarcopenia by hindering muscle maintenance (**Figure 4**).This highlights the critical importance of monitoring sarcopenia in CRC patients undergoing targeted therapies, as it not only affects nutritional and immune status but also impacts treatment efficacy and toxicity.

One of the innovative aspects of this research is the identification of the SYK gene as a risk factor for sarcopenia development in patients treated with fruquintinib. The SYK is a non-receptor protein tyrosine kinase involved in immune signaling and tumor metastasis initiation (47). Meanwhile, the SYK is a cytoplasmic enzyme that links immune cell receptors to intracellular signaling, crucial for adaptive and inflammatory immune responses (48, 49). The SYK gene exhibits dual functionality, acting as both a promoter and a suppressor of tumorigenesis (50). SYK gain-of-function variants found in patients with immune deficiency and systemic inflammation boost immune signaling and elevate inflammatory cytokines. A SYK-S544Y knock-in mouse model mirrored human disease symptoms, which were partially relieved by SYK inhibitors (51). These findings emphasize the pivotal role of SYK in amplifying inflammatory and immune responses, and underscore the potential of targeting SYK as a fundamental therapeutic strategy for the management of related disorders. However, no studies have identified a correlation between the SYK gene and cancer-associated sarcopenia, indicating a need for further in-depth investigation in future research. Our study found that sarcopenia is closely related to inflammation and immune responses in advanced CRC patients treated with fruquintinib. We identified SYK as a risk gene for fruquintinib-induced sarcopenia through MR analysis. This discovery offers a novel perspective on the genetic basis of sarcopenia in cancer patients and may guide future precision medicine approaches. From a clinical perspective, our findings underscore the importance of early detection and management of sarcopenia, as addressing this condition may enhance the efficacy of treatments such as fruquintinib and mitigate associated adverse side effects. By improving nutritional status and enhancing immunity, nutrition intervention can help alleviate symptoms and improve the prognosis for patients with CRC (52, 53). Thus, we can conclude that nutritional interventions and physical rehabilitation should be integrated into the standard care of CRC patients, especially those at risk for sarcopenia, to optimize outcomes.

This study has several limitations, including its retrospective design, which limits causal inference. Further prospective studies are needed to confirm these findings and explore the underlying mechanisms. Additionally, factors such as muscle strength, physical activity, and exercise interventions were not evaluated, and should be considered in future research. Further validation of the SYK gene in larger cohorts is also needed.

In conclusion, sarcopenia may be an important prognostic factor for patients with advanced colorectal cancer undergoing fruquintinib targeted therapy. Early identification and management of sarcopenia could potentially enhance treatment outcomes and reduce the risk of complications in these patients.

## Data Availability

The original contributions presented in the study are included in the article/**Supplementary Material**. Further inquiries can be directed to the corresponding authors.
