# Correction: The impact of sarcopenia on prognosis and fruquintinib efficacy in advanced colorectal cancer: a retrospective and mendelian randomization study

**DOI:** 10.3389/fimmu.2025.1671178

**Published:** 2025-08-05

**Authors:** Frontiers Production Office

**Affiliations:** Frontiers Media SA, Lausanne, Switzerland

**Keywords:** sarcopenia, immune, nutrition, advanced colorectal cancer, fruquintinib, mendelian randomization study

Due to a production mistake, there was a mistake in **Table 3** and **Table 5** as published. The tables were swapped. The corrected **Table 3** and **Table 5** appear below.

**Table 3:** MR results of exposure factors and outcomes (left hand grip strength).

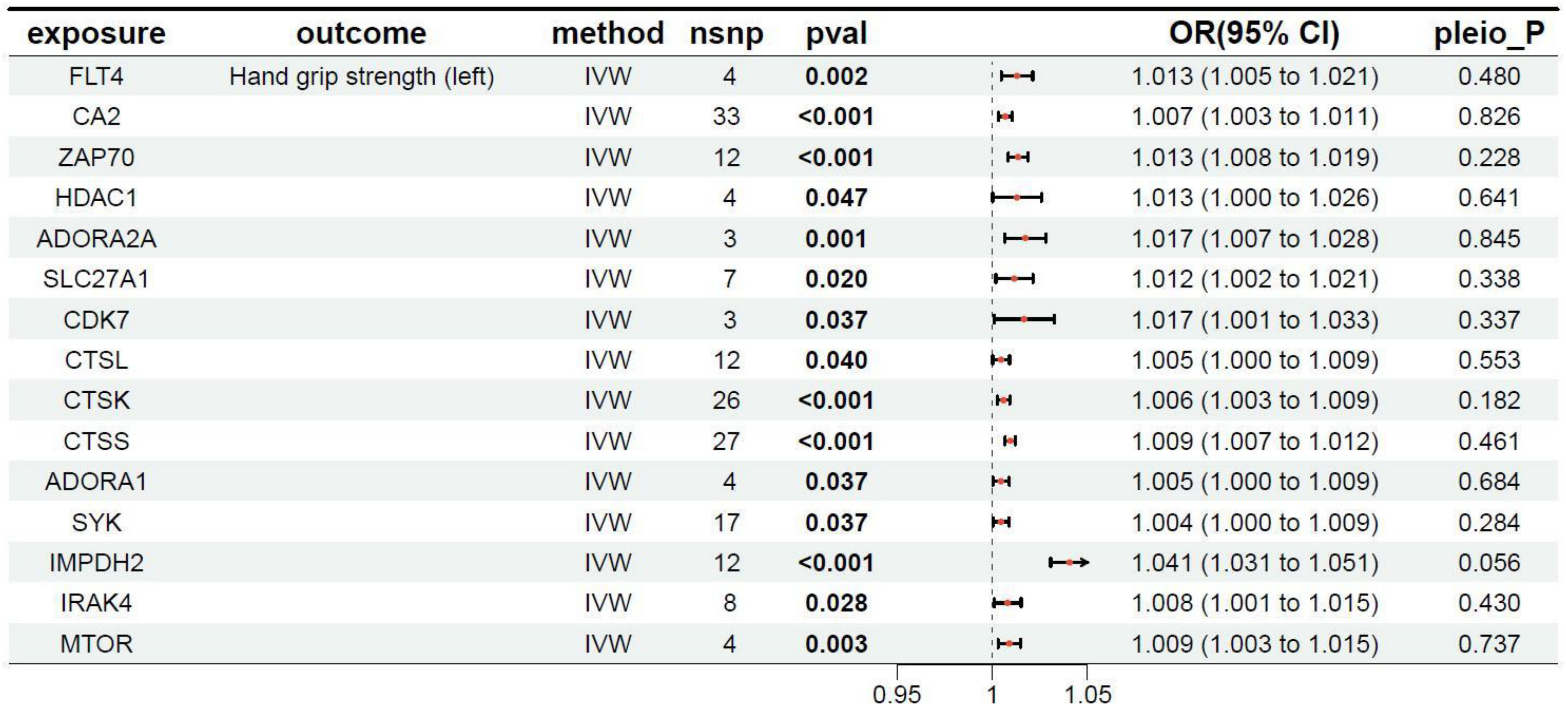

pavl: Significance; OR, Odds ratio, with the 95% confidence interval of the odds ratio in parentheses; pleio_P: Test for horizontal pleiotropy.

**Table 5:** MR results of exposure factors and outcomes (limb muscle mass).

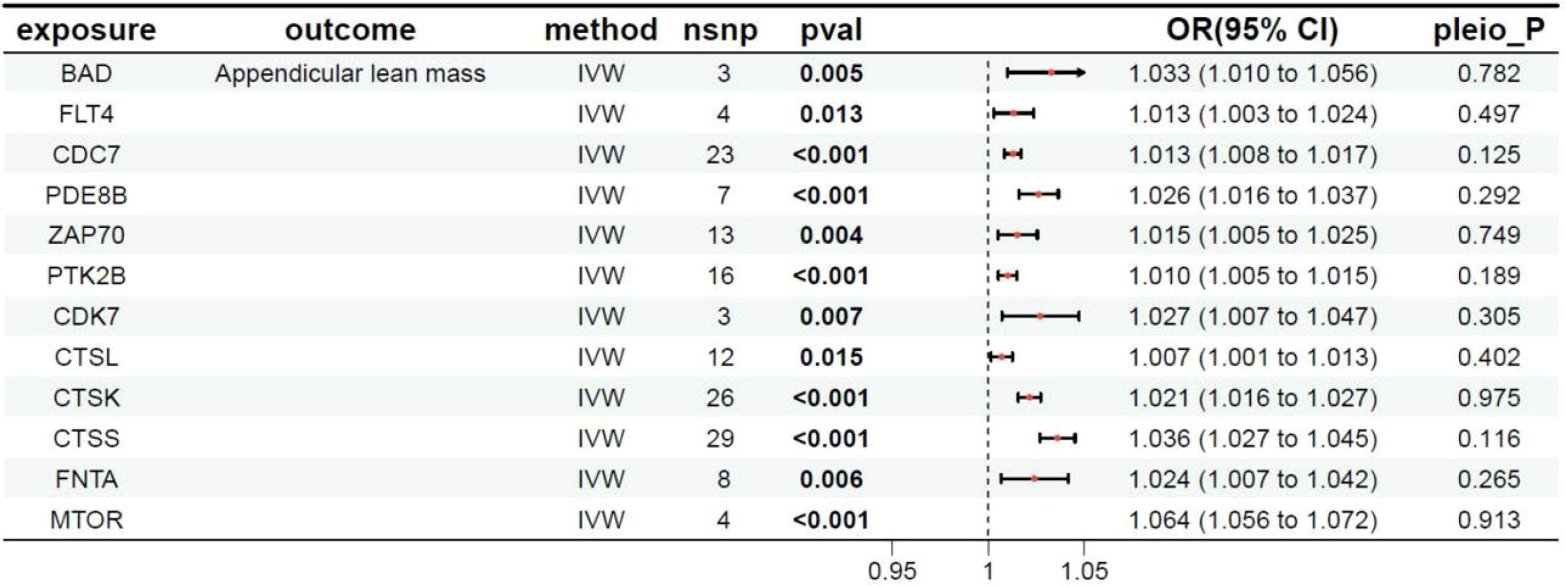

pavl: Significance; OR, Odds ratio, with the 95% confidence interval of the odds ratio in parentheses; pleio_P: Test for horizontal pleiotropy.

The publisher apologizes for the error and the original version of this article has been updated.

