# Supplementary figures and images for: The impact of sarcopenia on prognosis and fruquintinib efficacy in advanced colorectal cancer: a retrospective and mendelian randomization study
Source: Front Immunol. 2025 Jul 16;16:1582308. doi: 10.3389/fimmu.2025.1582308 (PMC12307208; doi:10.3389/fimmu.2025.1582308)

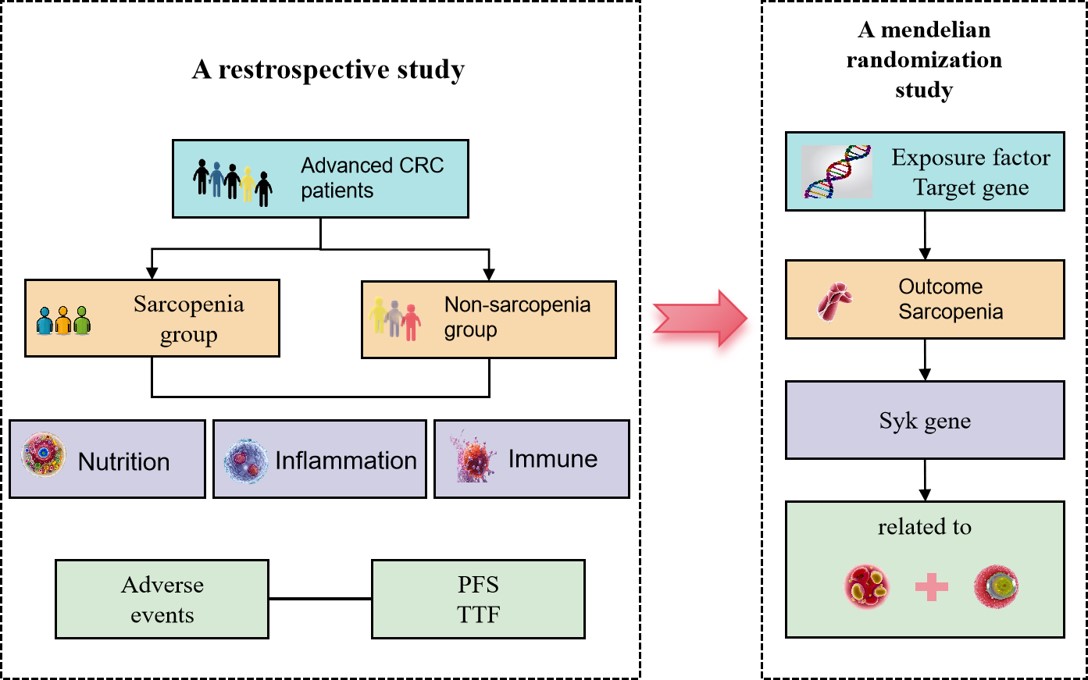

Supplement: Supplementary file 1 [file Image1.jpeg]
