# Supplementary material for: The impact of sarcopenia on prognosis and fruquintinib efficacy in advanced colorectal cancer: a retrospective and mendelian randomization study
Source: Front Immunol. 2025 Jul 16;16:1582308. doi: 10.3389/fimmu.2025.1582308 (PMC12307208; doi:10.3389/fimmu.2025.1582308)
Supplement: Supplementary file 2 [file DataSheet1.zip › Supplemental materials/Supplemental figures.pdf]

Figure S1

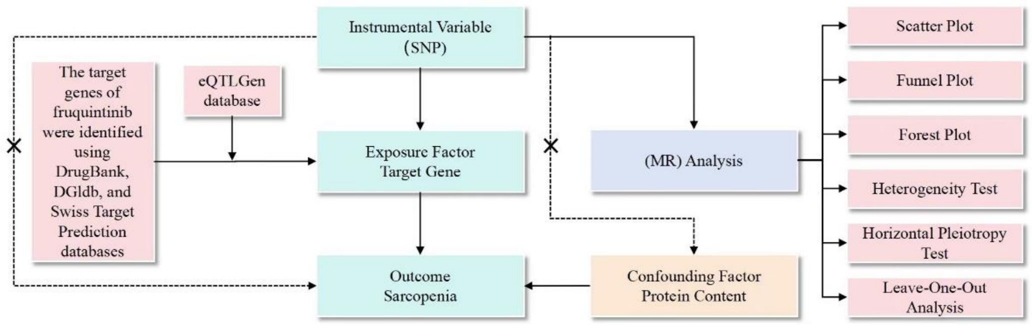

The study flowchart.

Figure S2

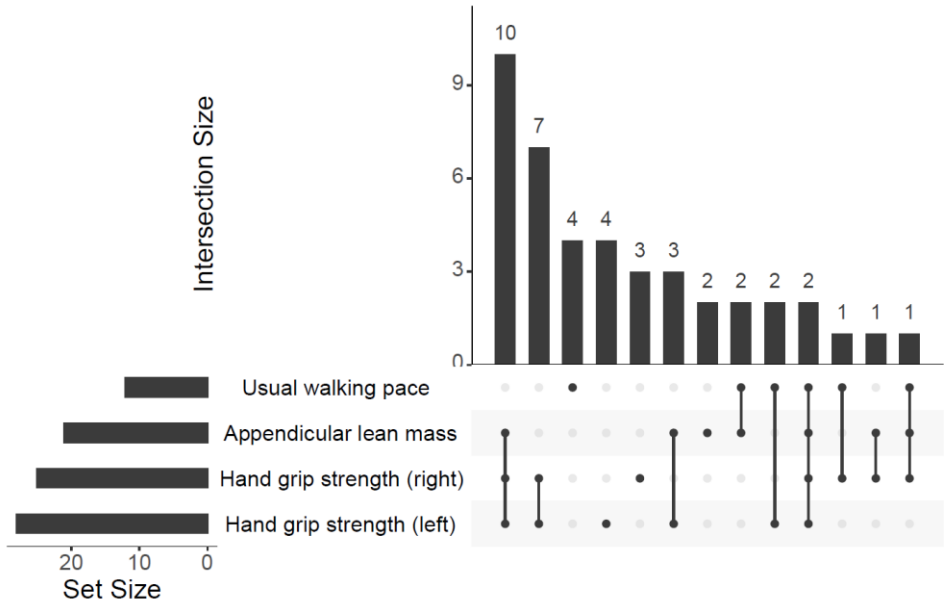

UpSet plot of significant results for the four outcome variables
